# Expression of TMPRSS4 in non-small cell lung cancer and its modulation by hypoxia

**DOI:** 10.3892/ijo.2012.1513

**Published:** 2012-06-12

**Authors:** TRI-HUNG NGUYEN, WILLIAM WEBER, EVIS HAVARI, TIMOTHY CONNORS, REBECCA G. BAGLEY, RAJASHREE McLAREN, PRASHANT R. NAMBIAR, STEPHEN L. MADDEN, BEVERLY A. TEICHER, BRUCE ROBERTS, JOHANNE KAPLAN, SRINIVAS SHANKARA

**Affiliations:** Genzyme Corporation, 49 New York Avenue, Framingham, MA 01701, USA

**Keywords:** TMPRSS4, lung cancer, carbonic anhydrase IX, hypoxia, serine protease

## Abstract

Overexpression of TMPRSS4, a cell surface-associated transmembrane serine protease, has been reported in pancreatic, colorectal and thyroid cancers, and has been implicated in tumor cell migration and metastasis. Few reports have investigated both TMPRSS4 gene expression levels and the protein products. In this study, quantitative RT-PCR and protein staining were used to assess TMPRSS4 expression in primary non-small cell lung carcinoma (NSCLC) tissues and in lung tumor cell lines. At the transcriptional level, TMPRSS4 message was significantly elevated in the majority of human squamous cell and adenocarcinomas compared with normal lung tissues. Staining of over 100 NSCLC primary tumor and normal specimens with rabbit polyclonal anti-TMPRSS4 antibodies confirmed expression at the protein level in both squamous cell and adenocarcinomas with little or no staining in normal lung tissues. Human lung tumor cell lines expressed varying levels of TMPRSS4 mRNA *in vitro*. Interestingly, tumor cell lines with high levels of TMPRSS4 mRNA failed to show detectable TMPRSS4 protein by either immunoblotting or flow cytometry. However, protein levels were increased under hypoxic culture conditions suggesting that hypoxia within the tumor microenvironment may upregulate TMPRSS4 protein expression *in vivo*. This was supported by the observation of TMPRSS4 protein in xenograft tumors derived from the cell lines. In addition, staining of human squamous cell carcinoma samples for carbonic anhydrase IX (CAIX), a hypoxia marker, showed TMPRSS4 positive cells adjacent to CAIX positive cells. Overall, these results indicate that the cancer-associated TMPRSS4 protein is overexpressed in NSCLC and may represent a potential therapeutic target.

## Introduction

Lung cancer is the second most common cancer in both men and women worldwide. According to the United States Cancer Statistics (USCS) for 2007 (the most recent for which the statistic is available), a total of 203,536 new cases of lung cancer were diagnosed and 158,683 deaths were reported (1). The lung cancer mortality rate outranks that of any other cancers. Lung cancer has been classified into two major types, small cell lung cancer (SCLC) and non-small cell lung cancer (NSCLC). NSCLC, characterized by aggressive growth and often metastasizes to distant lymph nodes and vital organs, comprises 80% of new cases of lung cancer diagnosed each year and is further sub-classified based on tumor histology into epidermoid or squamous cell carcinoma (SCC), adenocarcinoma, and large cell carcinoma. Treatments for lung cancer include surgery, chemotherapy, radiation therapy, and immunotherapy; however, these standard treatments are rarely curative. Therefore, new treatments and therapies are being developed, and biomarkers allowing early diagnosis are being sought (2–5).

Cell surface proteases play a major role in cancer progression and metastasis (6). In normal tissues, cell surface proteases are involved in regulating cellular activities, such as cell-cell interaction, adherence to matrix components, motility and homeostasis. Overexpression of these proteases alters the cell surface:matrix interaction and, subsequently, cell morphology. TMPRSS4, a type II transmembrane protein serine protease belongs to a family of membrane-type serine proteases (MT-SP), discovered by differential gene analysis for pancreatic cancer markers (7). The full-length cDNA was cloned and originally designated as TMPRSS3 (8), a name previously assigned to a closely related protein located on chromosome 21q22.3. TMPRSS4 was subsequently mapped to chromosome 11q23.3 and has been shown to have two alternate splice variants producing transcripts of 2.12 and 1.98 kb. Murine TMPRSS4 is also called CAP2 based on the amino acid sequence identity between the human protein and the mouse ortholog. Murine TMPRSS4 belongs to a family of channel activating proteins comprised of CAP-1, -2, and -3. Human TMPRSS4 and CAP-2 share 77.2% identity in amino acid sequences. CAP-2 has been described as activating the epithelial sodium channel (eNAC), a non-voltage-gated sodium channel that regulates the extracellular sodium concentration, and in turn, balances blood volume and pressure by controlling fluid re-absorption (9,10).

Since the cloning of the human TMPRSS4 gene, several studies have reported high expression of TMPRSS4 at the transcriptional level in pancreatic, colorectal, and thyroid cancers via Northern blot analyses, microarray gene-chips, and RT-PCR (11–15). Recent qPCR studies showed that high levels of TMPRSS4 message in NSCLC patients were associated with a poor prognosis (16). Studies of biological activity have reported that elevated TMPRSS4 expression induced epithelial to mesenchymal transition (EMT) of cancer cells and promoted metastasis (17,18). In addition, siRNA knockdown of TMPRSS4 in cancer cell lines and in metastatic potential mouse model, reduced cell invasion and migration, thus implying a role for TMPRSS4 in metastasis (16,19). Further analysis of TMPRSS4-mediated signaling in cancer cells suggested that multiple downstream signaling pathways are activated including focal adhesion kinase (FAK) and extracellular signal regulated kinase (ERK) resulting in the downregulation of E-cadherin and induced expression of integrin α5, a critical molecule implicated in tumor cell invasion, migration and tumor progression (18).

Most studies to date have focused on TMPRSS4 mRNA rather than protein levels and endogenous protein expression of TMPRSS4 in normal lung and NSCLC cells has not been examined. The general lack of information on TMPRSS4 protein expression is due, in part, to difficulty in generating reagent antibodies that are suitable for detecting denatured and native TMPRSS4 protein. Here, we successfully generated a TMPRSS4-specific rabbit polyclonal antibody and, in conjunction with qPCR, immunohistochemistry (IHC) and immunoblot analyses, explored TMPRSS4 expression and its potential as a therapeutic target in non-small cell lung cancers. Using human lung cancer cell lines and tumor xenograft models, we also investigated the role of hypoxia in modulation of TMPRSS4 protein expression in the tumor microenvironment.

## Materials and methods

### Cell lines and materials

Normal and tumor lung tissues were obtained from Biochain (Hayward, CA). Cell lines were obtained from ATCC (Manassa, VA) unless specified. All cells were maintained in DMEM-high glucose medium with 10% fetal bovine serum (Life Technologies, Carlsbad, CA). TMPRSS4 cDNA in a pENTR™221 vector was purchased from Life Technologies. Female Balb/c nu/nu mice were obtained from Harlan Laboratories (Boston, MA).

### Quantitative RT-PCR analysis

Total RNA from cell pellets and lung tissues was extracted using the RNEasy kit (Qiagen, Valencia, CA) with DNase treatment to remove genomic DNA. cDNA was produced using the High-Capacity cDNA Archive Kit (ABI, Foster City, CA). Primers and minor groove binder (MGB) probe were designed using the ABI’s Primer Express1.5. Primers were synthesized by Integrated DNA Technologies (Coralville, IA); forward primer: 5′-CCTGGCGAGTATCATCATTGTG-3′, reverse primer: 5′-CCCGCAGAGGAAGTAGTATTTATCC-3′. The probe was labeled at the 5′-end with 6-carboxyfluorescein reporter dye and on the 3′-end with non-fluorescent quencher. 18S rRNA was used as an internal standard for multiplex PCR (VIC/MGB probe, primer). The experiment was carried out in duplicate multiplex PCR reactions using 25 ng cDNA, the 2X Taqman^®^ Universal Master Mix, 900 nM gene of interest (GOI) primers, 250 nM GOI probes, 75 nM 18S rRNA primers, and 200 nM 18S rRNA probe. The amplification was performed using the ABI 7900HTS, in which the protocol was set as follows: 50°C, 2 min; 95°C, 10 min; annealing and extension at 95°C for 15 sec, 60°C for 1 min (40 cycles), respectively. To ensure lack of competition between the GOI and 18S rRNA, a validation experiment was performed where primers and probes for TMPRSS4 and 18S rRNA were used together and separately. Plasmid vector encoding TMPRSS4 was serially diluted to detect differences in mRNA over a wide dynamic range. The data were analyzed using the ABI SDS2.1 software. The data from duplicate wells were averaged. The comparative CT method was used to calculate relative levels of these transcripts in tumors compared with normal controls according to ABI User Bulletin no. 2. The average values were graphed using GraphPad Software Prism 5.0 and one-way ANOVA analysis of variance to test for statistical significance.

### Cloning TMPRSS4 cDNA

TMPRSS4 cDNA in a pENTR™221 vector was transferred to pcDNA6.2/V5-DEST mammalian expression vectors containing the Tag-On-Demand System (Invitrogen’s Gateway system) by performing the LR recombination reaction according to the Invitrogen protocol. The resulting vector was sequenced to confirm the correct cDNA and in-frame to the amber stop codon. Plasmid DNA was amplified in bacterial culture and was isolated using GenElute HP endotoxin-free plasmid maxiprep (Sigma, St. Louis, MO).

### Generation of rabbit polyclonal anti-TMPRSS4

A peptide containing amino acids 104–119 in the scavenger receptor cysteine-rich (SRCR) domain of TMPRSS4 was synthesized (Pacific Immunology, Ramona, CA). A cysteine was added at the carboxyl-terminal to facilitate the conjugation to keyhole limpet hemocyanin (KLH) via EDC-mediated conjugation (20). Peptide-KLH complex was used to immunize two New Zealand white rabbits with 100 *μ*g of protein in equal volumes of PBS and Freund’s complete adjuvant. Rabbits were boosted every three weeks with peptide-KLH complex in incomplete Freund’s adjuvant. Rabbit sera were collected prior to immunization and every two-weeks after the initial immunization. ELISA was performed to determine serum titers against the synthetic peptide. The positive sera were pooled and stored at −20°C. For affinity purification of the polyclonal anti-TMPRSS4, the synthetic peptide was conjugated to CNBr-activated Sepharose 4B beads (GE Healthcare Life Sciences, Piscataway, NJ). Prior to loading on the column, the pooled rabbit serum was diluted 1:1 with PBS, the sample was applied to the column, and the absorbance of the eluent was monitored at 280 nm. The column was washed extensively with PBS until A_280_ values reached baseline. Bound antibodies were eluted with 0.2 M glycine, pH 1.85, until the absorbance reached baseline and immediately neutralized with 1 M Tris-HCl, pH 8.5. The eluate was concentrated to 1–2 mg/ml and dialyzed against PBS with 0.02% sodium azide. Purified polyclonal anti-TMPRSS4 was aliquoted and stored at 4°C.

### Flow cytometric analysis

Cells were cultured either under normoxic condition (atmospheric O_2_) or for 48 h in a hypoxic chamber where the O_2_ level was maintained at 0.7% with 5% CO_2_. Hypoxic cells were dissociated with Versene (Life Technologies) and fixed in 4% paraformaldehyde prior to removing cells from the chamber. Cells under normoxic condition were dissociated similarly. Normoxic and hypoxic cells (1×10^6^) were then washed in Hanks’ balanced salt solution (HBSS) and resuspended in staining buffer (HBSS, 1% BSA, 0.1% sodium azide) before incubating with anti-TMPRSS4 antibodies for 30 min followed with F(ab′)_2_-FITC conjugated anti-Fc (20 *μ*g/ml). Washing was performed between antibodies. In the final step, cells were washed in HBSS, and flow cytometry analysis was performed on Beckman Coulter FC500. Data were analyzed via FlowJo (Tree Star, Inc., Ashland, OR).

### Immunoblot analyses

COS-1 cells were transfected with TMPRSS4-pcDNA6.2/V5 plasmid via Lipofectamine-2000 (Life Technologies). Twenty-four h post-transfection, cells were either left alone for expression of TMPRSS4 protein or treated with 100:1 pfu of adenovirus carrying the Tag-On-Demand amber stop codon suppressor (Life Technologies) for expression of TMPRSS4-V5 tagged fusion protein. At 48 h post transfection, cells were lysed in RIPA buffer containing protease inhibitors (Sigma). Cell lysates were electrophoresed on 4–12% SDS-PAGE gels with MOPS running buffer and then transferred onto nitrocellulose membranes. The membranes were incubated in either 1 *μ*g/ml of rabbit polyclonal anti-TMPRSS4 or mouse anti-V5 tag (Life Technologies) followed by a peroxidase-conjugated secondary antibody. A washing step with PBS-Tween-20 (0.05%) was performed after the change of antibodies and at the final step. The immunopositive protein bands were detected by Biomax-MS film via chemiluminescence substrate (Thermo Scientific, Rockfort, IL). Mouse anti-GAPDH was used to confirm that equal amounts of protein were analyzed in each sample.

For lung tumor tissues, lysates were prepared at 3.75 *μ*g/ml in NuPAGE buffer containing 1 mM DTT and denatured by heating at 80°C for 10 min. Proteins were analyzed by immunoblotting with rabbit polyclonal anti-TMPRSS4 as described above.

### Immunohistochemistry

A tissue array containing 101 formalin-fixed, paraffin-embedded tissue cores of lung SCC, adenocarcinomas, and normal non-neoplastic lung tissues from multiple donors was used (Phylogeny Inc., Columbus, OH). Tissue sections were deparaffinized in xylene, and antigen retrieval was carried out with proteolytic epitope retrieval (pepsin, BioCareMedical, Concord, CA) at room temperature (RT) for 15 min. Endogenous peroxidase was quenched with peroxidase block (Dako, Carpinteria, CA). The tissues were blocked with 10% goat serum (Sigma) for 10 min followed by exposure to rabbit polyclonal anti-TMPRSS4 at 10 *μ*g/ml for 60 min at RT. The rabbit polyclonal anti-TMPRSS4 was detected with biotinylated anti-rabbit IgG (H+L) (Jackson ImmunoResearch, West Grove, PA) for 20 min, and then ABC Elite (Vector Labs, Burlingame, CA) for an additional 20 min. The chromagen DAB (diaminobenzidine, Dako) was added to the tissue for 5 min as peroxidase substrate. The slides were rinsed in water, counterstained with hematoxylin, dehydrated in graded ethanol, cleared in xylene, and then mounted with permount. The level of TMPRSS4 expression was graded by an American College of Veterinary Pathologists board certified pathologist on a scale of 0–4 as follows: 0, no staining; 1, faint multifocal staining of cells; 2, diffuse, faint or moderate staining of cells; 3, multifocal, intense staining admixed with moderate staining of cells; 4, diffuse and intense staining of cells. Some specimens with a staining pattern falling between two score values were given 0.5 values.

Frozen tissue sections of lung squamous cell carcinoma sections were thawed to RT, fixed in 10% Zn/formalin (Electron Microscopy Sciences, Hatfield, PA) for 10 min, blocked with 10% goat serum, then incubated with a combination of rabbit polyclonal anti-TMPRSS4 and mouse monoclonal anti-CAIX (R&D Systems, Minneapolis, MN). For negative controls, sections were incubated with rabbit and mouse isotype controls. Following incubation with the primary antibodies, sections were incubated with Cy3 or Cy2-conjugated goat anti-rabbit IgG (H+L) and Cy2 or Cy3-conjugated goat anti-mouse IgG (H+L). After sections were washed in PBS, antifade mounting medium containing DAPI (4′,6-diamidino-2-phenylindole, dihydrochloride) (Life Technologies), was added to the slides prior to coverslipping.

### Generating xenograft tumors

Tumor cell lines were cultured and maintained in log phase. The day of injection (day 0), H358 and A549 cells were harvested by dissociation with trypsin, followed by 2 washes in PBS. Three female (*nu/nu*) nude mice were injected at two sites subcutaneously with a total of 2–4 million cells per mouse. Tumor volume was measured over time with calipers. Tumors were collected when they reached ∼250 mm^3^ in volume. Tumor tissues were fixed in 10% neutral buffered formalin at 4°C overnight. The next day, tumor tissues were removed from the fixative and washed with PBS followed by paraffin embedding and sectioning. Tissue sections were stained with rabbit anti-TMPRSS4 antibodies as described above. All animal studies were approved by Genzyme Institutional Animal Care and Use Committee and conducted in Genzyme Association for Assessment and Accreditation of Laboratory Animal Care accredited facility.

## Results

### TMPRSS4 mRNA upregulation in non-small cell lung carcinomas and in lung cancer cell lines

**Figure 1:**
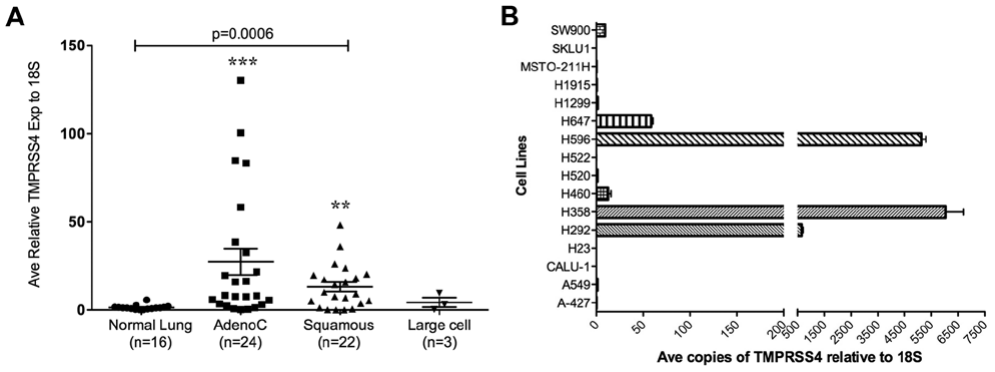
TMPRSS4 expression in NSCLC tissue specimens and lung cancer cell lines. (A) Quantitative RT-PCR was performed on 49 lung tumors and 16 normal donor tissues. For each sample, qPCR was performed in duplicate, and the average values plotted relative to 18S rRNA. One-way ANOVA analysis of variance was used with Kruskal-Wallis test for all four groups (adeno-, squamous, large cell carcinomas, and normal) (p=0.0006) followed by Dunn’s multiple comparison test with 99% confident intervals for normal vs adenocarcinomas (***), and normal vs squamous cell carcinomas (**). Bars represent the mean value for each group. ^**^p<0.01; ^***^p<0.001. (B) qPCR was performed on lung cancer cell lines. Total RNA was isolated from 16 lung cancer cell lines. qPCR was performed, and the average values from duplicate samples were normalized against 18S rRNA. The average relative copies of TMPRSS4 are plotted.

A total of 49 human NSCLC tumor specimens including 24 adenocarcinomas, 22 squamous and 3 large cell carcinomas were obtained. Normal lung tissue from 16 donors was also obtained. Total RNA was isolated from each sample, and quantitative RT-PCR analysis (qPCR) was performed in duplicate for each sample. The average relative TMPRSS4 expression was calculated in comparison with 18S ribosomal RNA and plotted by tumor subtype. TMPRSS4 transcript was highly expressed in adenocarcinomas and squamous cell carcinomas, while expression in the normal tissues was negligible (Fig. 1A) and only low levels were seen in large cell carcinomas. Statistical analysis was performed comparing the mean values for NSCLC samples to those of normal samples via one-way ANOVA analysis of variance with the Kruskal-Wallis test, followed by a Dunn’s multiple comparison tests. Differences in TMPRSS4 mRNA levels were statistically significant (p=0.0006) with 99% confidence intervals for Kruskal-Wallis test, and Dunn’s post-test displayed p<0.01 for normal vs. adenocarcinoma and normal vs. squamous cell carcinomas. In both types of tumor, >70 and 59% of samples showed 3- and 5-fold or greater TMPRSS4 mRNA expression than normal lung tissue, respectively (Table I). The average value for large cell carcinomas, even though few samples were analyzed, was higher than for the normal lung samples, but considerably lower than for adenocarcinomas and squamous cell carcinomas, and statistically not different from normal lung tissue. These results indicate that TMPRSS4 message is significantly upregulated in most primary NSCLC tumor tissues.

We next examined TMPRSS4 message in human lung cancer cell lines in culture. TMPRSS4 qPCR was performed on 16 lung cancer lines and variable numbers of TMPRSS4 transcripts were detected in 6 of 16 lung cancer lines. The H358 and H596 NSCLC cell lines expressed the highest levels with >4500 copies relative to 18S, followed by the H292 cell line with 500 copies, and fewer copies in the H647, H460, SW900 lines and A549 (Fig. 1B). These results indicate that while TMPRSS4 message is significantly upregulated in a majority of NSCLC primary tumor tissues, it is not widely expressed in lung cancer cell lines.

### Generation of rabbit polyclonal anti-TMPRSS4

**Figure 2:**
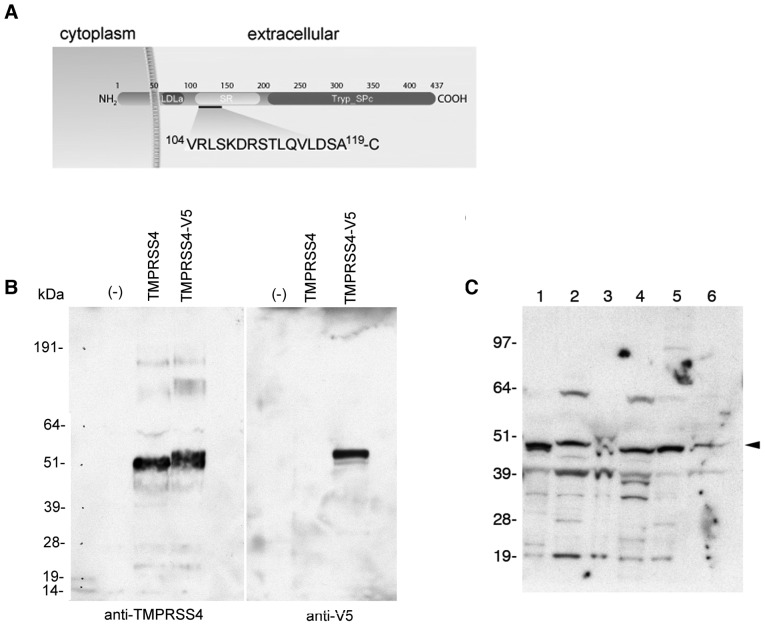
(A) Schematic drawing of TMPRSS4 domains and the peptide sequence used to raise rabbit antibodies. Figure is not drawn to scale. LDLa, low-density lipoprotein receptor domain class A; SRCR, scavenger receptor cysteine-rich; Tryp_Spc, trypsin-like serine protease. (B) Immunoblotting of affinity-purified rabbit antibodies against TMPRSS4 fusion protein expressed in COS-1 cells. COS-1 cells were transfected with TMPRSS4-pcDNA6.2/V5 for TMPRSS4 expression. Adenoviruses carrying Tag on-demand amber stop codon suppressor were added to generate TMPRSS4-V5 fusion protein expression. Untransfected COS-1 cells were performed in parallel and labeled as (−). Each lane of the SDS-PAGE received 50 *μ*g of total protein from the cell lysate, and the immunoblotting was performed with rabbit polyclonal anti-TMPRSS4 (1:1000 dilution). (C) Immunoblotting of rabbit polyclonal anti-TMPRSS4 against primary lung tumor samples. Lysates from six squamous cell carcinoma samples were subjected to SDS-PAGE followed by immunoblotting with rabbit polyclonal anti-TMPRSS4. Each lane contained 75 *μ*g of total protein from tumor lysate. The major protein band, indicated by an arrowhead, is TMPRSS4.

Rabbit polyclonal anti-TMPRSS4 antibodies were generated against a synthetic peptide covering amino acid position 104–119 within the extracellular scavenger receptor cysteine-rich (SRCR) domain of human TMPRSS4 (Fig. 2A). This region was selected based upon a high antigenicity index according to a hydropathy plot (8,21) and low similarity to other TMPRSS family members. To confirm the specificity of rabbit antibodies against TMPRSS4, COS-1 cells were transiently transfected to express TMPRSS4-V5 tagged fusion protein or TMPRSS4 alone as described in Materials and methods. Total cell lysates were subjected to SDS-PAGE electrophoresis followed by immunoblotting with anti-TMRPSS4 and anti-V5 antibodies (Fig. 2B). TMPRSS4 alone migrated on a denatured SDS-PAGE gel at ∼51 kDa relative to standard markers, while TMPRSS4-V5 fusion protein migrated at a slightly higher molecular weight as a result of the V5 fusion. Faint immunopositive bands at both higher and lower molecular weights were observed with rabbit polyclonal anti-TMPRSS4 suggesting that TMPRSS4 may form dimers and trimers with corresponding molecular weights ∼100 and 150-kDa, respectively, while lower molecular weight bands may reflect proteolysis associated with TMPRSS4 activity. No staining was observed for untransfected COS-1 cells further demonstrating the specificity of the antibodies. To demonstrate that affinity-purified rabbit polyclonal anti-TMPRSS4 can detect TMPRSS4 in lung tumor specimens, six squamous cell carcinoma lysates were subjected to immunoblotting. A dominant band at ∼51 kDa was observed with labeling of some lower molecular weight bands (Fig. 2C). The 51-kDa protein band failed to migrate uniformly in all samples, another indication of proteolysis or post-translation modification of TMPRSS4 in the tumor specimens. High molecular weigh bands corresponding to the 64 kDa marker were observed in three samples suggesting splice-variants of TMPRSS4 in selected primary tumor tissues. Furthermore, no protein band from lung tumor specimens was observed in a blocking experiment, in which anti-TMPRSS4 antibodies were incubated with TMPRSS4 (104–119) peptide before exposing to the blot (data not shown). These results demonstrated that our affinity purified rabbit polyclonal anti-TMPRSS4 recognizes TMPRSS4 expressed in transfected cells and in primary lung tumors.

### TMPRSS4 protein overexpression in human non-small cell lung carcinomas

**Figure 3:**
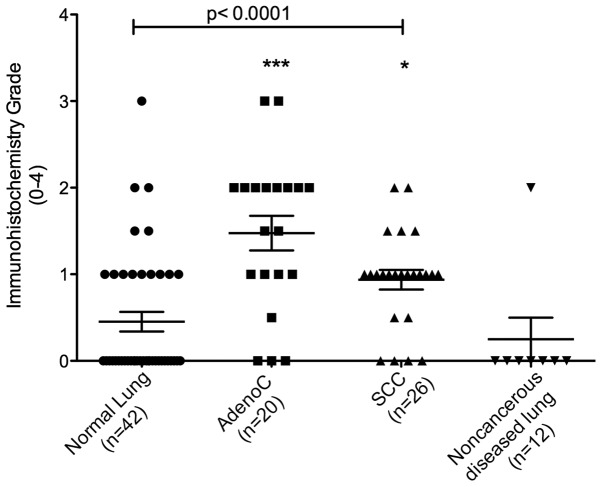
Immunohistochemical grades of normal lung and NSCLC tissue specimens stained with rabbit polyclonal anti-TMPRSS4. Immunohistochemical staining intensity for each specimen was scored with grades 0–4 (see Materials and methods). Each symbol represents a tumor from an individual patient while the horizontal bars are group mean scores. Statistical comparisons between groups were done using Kruskal-Wallis test (p<0.0001) followed by Dunn’s multiple comparison tests, normal vs adenocarcinoma (***) and normal vs squamous cell carcinoma (^*^), no significance for normal vs noncancer diseased lung. ^*^p<0.05; ^***^p<0.001.

**Figure 4:**
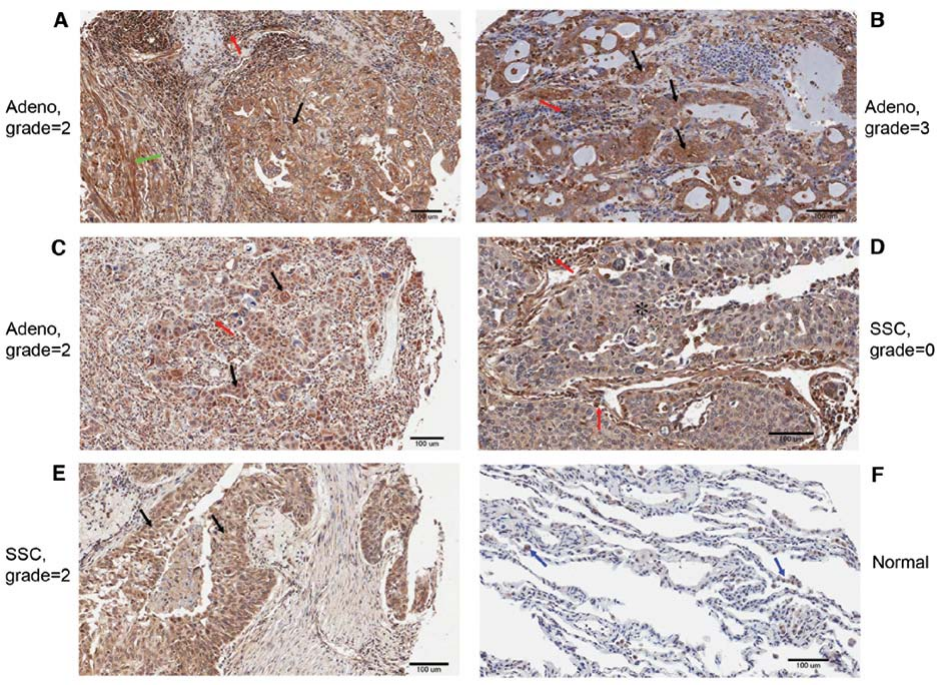
Representative immunohistochemical analysis of rabbit polyclonal anti-TMPRSS4 against primary NSCLC tissue specimens. Positive immuno-reactivity is indicated by the brown staining. Black arrow, immunopositive tumor cells; red arrow, immunopositive lymphocytes, macrophages, and alveolar macrophages; green arrow, immunopositive smooth muscle. Adenocarcinomas (A–C) exhibited greater immunoreactivity with lower background staining compared to squamous cell carcinomas (SCC) of the lung (D and E). Infiltrating lymphocytes and alveolar macrophages exhibited mild to moderate immunoreactivity with the antibody. (D) SCC that does not stain (^*^) while the infiltrating inflammatory cells are immunopositive. (F) Lack of staining in normal lung tissue except normal alveolar septa (blue arrow) that were immunopositive.

To assess TMPRSS4 protein expression in human lung tumor tissue sections, we performed immunohistochemistry (IHC) using affinity-purified rabbit polyclonal anti-TMPRSS4 on paraffin-embedded lung tumors and corresponding normal lung tissue specimens. A total of 100 tissue specimens were examined including 20 adenocarcinomas, 26 squamous cell carcinomas, 42 normal lung tissue samples, and 12 tissue samples from non-cancer diseased lungs (congested lung, collapsed lung, hyperplasia and pulmonary edema). Each tissue section was analyzed and assigned an IHC intensity score (0–4) by a pathologist. Overall, adenocarcinoma and squamous cell carcinoma tissues exhibited significantly higher mean IHC scores than the matched normal lung tissues (Fig. 3). The mean IHC scores of the squamous cell carcinomas were lower than adenocarcinomas. There was a wide variation in staining intensity in both types of lung tumors (Fig. 4). Interestingly, 50% of the lung adenocarcinomas demonstrated moderate to marked TMPRSS4 staining (IHC score of ≥2) (Fig. 4A–C), while only 20% of the lung squamous cell carcinomas showed similar staining intensity (Fig. 4D and E). Rabbit polyclonal anti-TMPRSS4 also stained resident alveolar macrophages, infiltrating inflammatory cells (i.e., lymphocytes and macrophages), and occasional alveolar septae (Fig. 4F). In the normal lung tissue specimens, ciliated epithelial cells lining the bronchioles (when present) demonstrated mild to moderate cytoplasmic staining (data not shown). No statistically significant variations were found for TMPRSS4 staining in tissue sections from congested lung, chronic pneumonitis with alveolar epithelium hyperplasia and collapsed lung, and the IHC scores were similar to those of normal lung tissue. These results are consistent with the mRNA expression data described in Fig. 1 indicating that TMPRSS4 expression is overall elevated in lung cancers compared to normal and non-cancer diseased lung tissues.

### Tumor microenvironment-dependent expression of TMPRSS4 protein in human lung cancer cell lines

**Figure 5:**
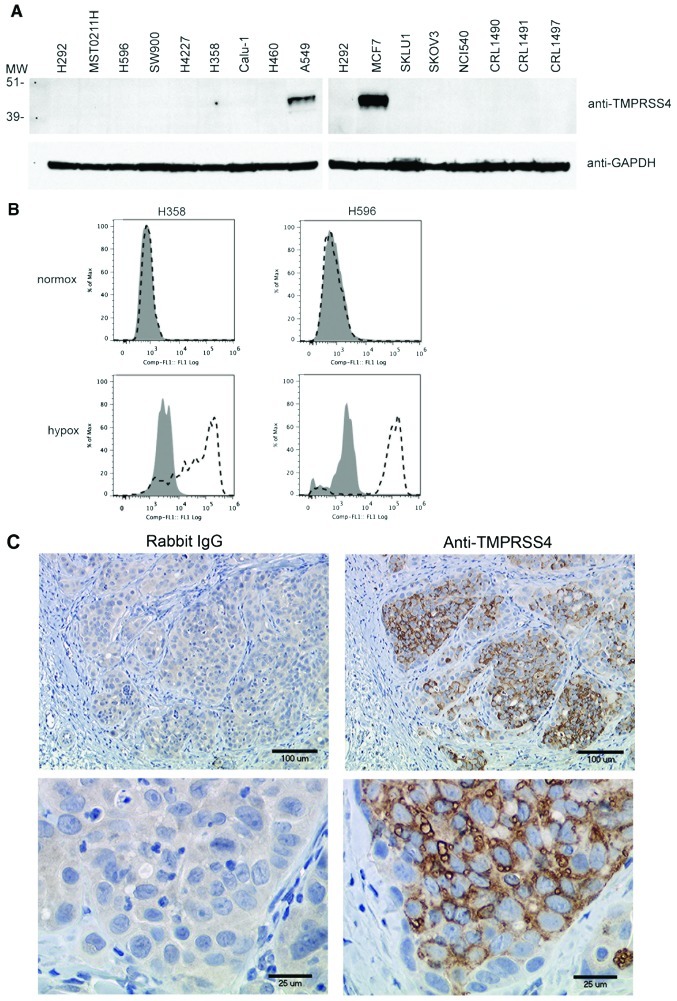
(A) Immunoblot analyses of cancer cell lysates for TMPRSS4 protein expression. Lysates from cancer cell lines along with three normal lung tissue samples (CRL1490, CRL1491 and CRL1497) were prepared in RIPA and SDS-PAGE buffers, and then subjected to SDS-PAGE and immunoblotting. The blots were probed with rabbit polyclonal anti-TMPRSS4 (1:1000 dilution) and then mouse anti-GAPDH (1:1000 dilution) to confirm that equal amounts of protein were being analyzed in each lane. Positive protein bands were detected with peroxidase-conjugated secondary antibodies and chemiluminescent substrate. (B) Lung cancer cell lines exposed to hypoxia. H358 and H596 cells were cultured under hypoxic (O_2_ <0.7%) or normoxic conditions for 48 h prior to staining with anti-TMPRSS4 for flow cytometry. Shaded, IgG isotype control; open dashed, anti-TMPRSS4 staining. (C) Tissue sections of xenograft tumors stained for TMPRSS4 with rabbit polyclonal anti-TMPRSS4. Nude mice were implanted with H358 human lung cancer cells to generate solid tumors, which were collected, fixed in formalin, and embedded in paraffin for sectioning. Tissue sections were stained with rabbit polyclonal anti-TMPRSS4 or with rabbit IgG antibodies, followed by biotinylated goat anti-rabbit as described in Materials and methods.

We next examined TMPRSS4 protein expression in a variety of human cancer cell lines in culture. TMPRSS4 messages were confirmed by conventional RT-PCR and were found consistent with qPCR data (Fig. 1B) (data not shown). When cell lysates were generated and analyzed by immunoblotting, only 2 cell lines stained positive for TMPRSS4 protein, the A549 lung cancer and MCF7 breast cancer lines (Fig. 5A). Anti-GAPDH was used as a loading control. As expected, no detectable TMPRSS4 protein was observed for SKLU1, MSTO211H, Calu-1 and normal lung tissues, which are lacking TMPRSS4 messages and consistent with Fig. 1. The absence of detectable TMPRSS4 protein in tumor lines with high levels of mRNA suggested the involvement of environmental factors in the control of mRNA translation. Hypoxia is known to prevail in the tumor micro-environment and was therefore tested as a potential regulator of TMPRSS4 protein expression. The H358 and H596 lung cancer cell lines were selected for these studies as they express high levels of TMPRSS4 transcripts but no detectable protein (Figs. 1B and 5A). Cells were cultured either under normoxic conditions or in a hypoxic chamber (O_2_ <0.7%) for 48 h. The cells were then stained with anti-TMPRSS4 antibody or an isotype control and were analyzed by flow cytometry (Fig. 5B). In both cell lines, TMPRSS4 protein was detected only when the cells were exposed to hypoxia and was undetectable in cells cultured under normoxic conditions.

To extend these *in vitro* studies to the *in vivo* setting, H358 cells were implanted into nude mice subcutaneously to generate tumors. When tumors reached a 250 mm^3^ volume, they were collected and serial tissue sections were stained with rabbit polyclonal anti-TMPRSS4. The H358 xenograft tumors stained intensely for TMPRSS4 by IHC with little or no background staining with an isotype control antibody (Fig. 5C). These results support the hypothesis that hypoxic conditions in the tumor environment may promote expression of TMPRSS4 protein.

### TMPRSS4 positive cells adjacent to CAIX positive cells in primary lung carcinomas

**Figure 6:**
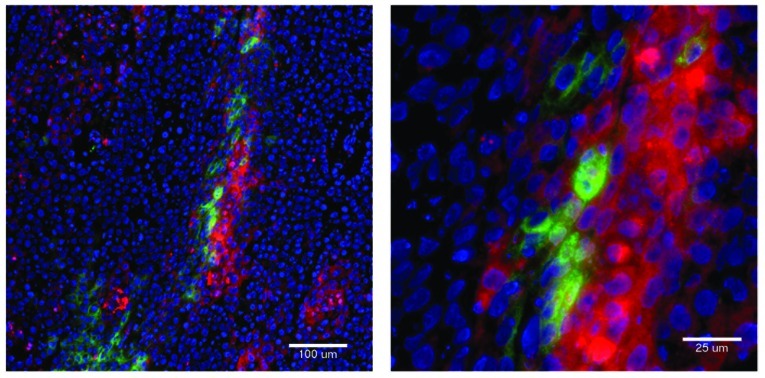
Lung squamous cell carcinoma stained for TMPRSS4 and CAIX. Frozen lung tumor tissue sections were co-stained with rabbit polyclonal anti-TMPRSS4 and mouse monoclonal anti-CAIX antibodies and then with secondary species-specific antibodies; Cy3-conjugated goat anti-rabbit (red) and Cy2-conjugated anti-mouse (green). Nuclei were stained with DAPI (blue). For negative controls, tissue sections were stained with rabbit and mouse IgG isotype antibodies.

Primary human lung carcinoma samples were used to determine whether expression of TMPRSS4 protein coincided with hypoxic regions within the tumor mass. Carbonic anhydrase IX (CAIX) was used as a hypoxia marker (26). Frozen tissue sections of human lung squamous cell carcinoma were stained with rabbit polyclonal anti-TMPRSS4 and mouse monoclonal anti-CAIX, and then with DAPI for nuclei. Strong staining for TMPRSS4 (Fig. 6, red color) and CAIX (green color) was observed. In most areas, CAIX positive cells were either surrounded by TMPRSS4 positive cells or vice versa indicating close proximity of TMPRSS4-expressing cells with the CAIX hypoxic marker and no coincident staining on the same cells. No expression of TMPRSS4 or CAIX was detected in the tumor stroma in agreement with Kivela *et al* and Juhasz *et al*(22,23). These results provide further support for an involvement of hypoxia in regulation of TMPRSS4 protein expression.

## Discussion

TMPRSS4 expression has been studied in several cancers at the transcriptional level but information is lacking on expression of the protein product. In this study, we examined TMPRSS4 mRNA as well as protein expression in human non-small cell lung cancer (24). Of the lung tumor specimens examined for TMPRSS4 transcripts, adenocarcinomas and squamous cell carcinomas showed significantly elevated levels of TMPRSS4 message compared with normal lung tissues similar to the recent report of Larzabal *et al*(16). Large cell lung carcinoma specimens had an average value higher than that of normal lung tissue, but the difference did not reach statistical significance, possibly due to the small number of samples tested. These results demonstrate that TMPRSS4 is over-expressed in a majority of NSCLC tumor and are in agreement with reports of elevated levels of TMPRSS4 message in pancreatic (8), thyroid (13), and metastatic cancers (15). To examine TMPRSS4 expression at the protein level, we generated a polyclonal rabbit antibody to a synthetic peptide from the extracellular domain of TMPRSS4 that has little similarity to other family members (e.g., TMPRSS2 and TMPRSS3) to decrease the probability of cross-reactivity. TMPRSS2 and TMPRSS3 extracellular domains display 38 and 36% amino acid identity, respectively, to the region of TMPRSS4 selected for immunization. The specificity of the rabbit polyclonal anti-TMPRSS4 antibody was confirmed by immunoblotting against COS-1 cells transduced with TMPRSS4 plasmid. TMPRSS4 protein expression in lung tumor samples was then evaluated by immunohistochemistry. Rabbit polyclonal anti-TMPRSS4 antibody produced positive staining in tumor tissue sections from lung adenocarcinomas and squamous cell carcinomas but not in sections from samples of normal lung tissue or congested, hyperplastic, pulmonary edema or collapsed lung tissue sections. These results indicate that the increase in TMPRSS4 transcripts observed in NSCLC tumors translates into an increased expression of the protein compared to normal or non-cancer diseased lung tissue.

TMPRSS4 expression was also evaluated in lung cancer cell lines, which are commonly used in experimental tumor models to study lung tumor biology and test potential therapeutic agents. In contrast to the elevated transcript levels found in a majority of primary NSCLC tumor samples, only six out of sixteen cell lines tested expressed TMPRSS4 transcripts by qPCR and conventional RT-PCR analyses (Fig. 1B) under normal culture conditions and, in a separate study, the majority of cell lines positive for TMPRSS4 message were found to be negative for protein expression (Fig. 5). This observed discrepancy in TMPRSS4 expression between primary tumor samples and tumor cell lines cultured *in vitro* suggested an influence of the environment on protein expression. Since the hypoxic conditions that commonly prevail in the tumor microenvironment are known to modulate gene expression, TMPRSS4 protein expression was evaluated under normoxic and hypoxic conditions in two tumor cell lines positive for TMPRSS4 mRNA (H358 and H596). Hypoxia was in fact found to induce TMPRSS4 protein expression on the surface of the cells as determined by flow cytometry. In addition, implantation of H358 tumor cells *in vivo* gave rise to tumors staining positive for TMPRSS4 protein (Fig. 5) suggesting that expression of TMPRSS4 within the tumor microenvironment may be promoted by hypoxic conditions as demonstrated in the metastatic hepatocyte carcinoma xenograft model in which elevated TMPRSS4 gene and protein product correlate to the HIF-1α expression level (25).

To further explore this possibility, primary human lung tumor specimens were co-stained for TMPRSS4 and CAIX, a known marker of hypoxia (26). Positive staining for TMPRSS4 and CAIX was observed on adjacent cells within the tumors with little or no coincident staining on the same cells. These results confirm expression of TMPRSS4 in hypoxic regions within tumors and support the contention that hypoxia may upregulate TMPRSS4 protein expression *in vivo*.

The significance of TMPRSS4 overexpression in cancers is not yet clear but is generally believed to promote tumor growth and metastasis. TMPRSS4 has been implicated in the induction of epithelial-mesenchymal transitions and in cancer metastasis (17,19). Overexpression of TMPRSS4 enzyme leads to the breakdown of extracellular matrix, and promotes invasion and migration of cancer cells in cell-based assays and induced the expression of integrin α5 (18). In *in vivo* studies, Jung *et al* demonstrated that more tumor cells distributed from the spleen to the liver in nude mice that were injected with SW480 cells engineered to overexpress TMPRSS4 compared to those injected with SW480 wild-type cells (17). In contrast, tail vein injection of H358 tumor cells knocked-down for expression of TMPRSS4 with shRNA resulted in decreased tumor metastasis to the lung (16). Other cell surface proteases have been shown to be overexpressed and to play a role in cancer metastasis, including members of the matrix metalloproteinase family and cell surface serine proteases (27,28). Overexpression of a cell surface protease has the potential to affect the extracellular matrix and to alter cell morphology thereby enhancing cell motility and invasiveness of distant organs.

TMPRSS4 substrates or interacting proteins in humans have not yet been identified. Recent study demonstrated in the co-transfected cell culture system that TMPRSS4 cleaves hemagglutinin protein expressed on the 1918 influenza virus and activates the virus infectivity (29). However, the mouse TMPRSS4 ortholog, CAP2, has been determined to activate the epithelial sodium channel (eNaC) (30–33). The expression pattern of eNaC includes the distal airways of the lung, the kidney, and the cochlea and is similar to the expression pattern of CAP2. ENaC has been described as a heterotetrameric protein comprised of ααβγ homologous subunits [reviewed by Rossier, Planes and Caughey and Matsushita *et al*(34–36)]. A δ-subunit of eNaC has also been described in pancreatic duct and in human melanoma (37–39). It has been proposed that murine TMPRSS4/CAP2 is expressed as a proenzyme, which can be stimulated to autocleave and, in turn, activate the eNaC channel by cleaving the gamma subunits (33). The regulation of eNaC is under tight control of the TMPRSS4/CAP2 protease and vice versa; however, the signal(s) involved in triggering TMPRSS4/CAP2 activity remain to be identified. A role for human TMPRSS4 interaction with eNaC in the tumor microenvironment has not been explored and represents an intriguing possibility. For example, hypoxia has been reported to cause a decrease in eNaC expression (40,41) leading to alveolar fluid accumulation (40) as is often observed in lung cancer patients. It is conceivable that hypoxia-induced downregulation of eNaC in the lung tumor microenvironment may trigger a compensatory increase in TMPRSS4 expression although this remains to be determined.

In conclusion, our results demonstrate the previously undescribed overexpression of TMPRSS4 in NSCLC at both the mRNA and protein levels. In addition, our findings suggest that expression of TMPRSS4 in the tumor microenvironment is regulated by hypoxia. The exact function and biological significance of TMPRSS4 overexpression remain to be fully characterized. Nonetheless, the consistent overexpression of TMPRSS4 at the gene level may represent a useful diagnostic or prognostic marker for lung cancer as suggested by Larzabal *et al* and as described by Kebebew *et al* in the context of thyroid neoplasm (42). At the protein level, TMPRSS4 may represent a potential target for antibodies or small molecule inhibitors of TMPRSS4 enzymatic activity for the treatment of NSCLC.

## Figures and Tables

**Table I:** Quantitative RT-PCR analysis of TMPRSS4 expression in lung tumor tissues relative to normal tissues.

Lung classification	Total samples tested	>3-fold	>5-fold	>10-fold	>20-fold
Adenocarcinoma	24	70.8%	62.5%	45.8%	29.2%
Squamous cell carcinoma	22	72.7%	59.1%	45.5%	9.1%
Large cell carcinoma	3	33.3%	33.3%	0.0%	0.0%

## Abbreviations:

NSCLC: non-small cell lung cancer
